# Trehalose Metabolism: From Osmoprotection to Signaling

**DOI:** 10.3390/ijms10093793

**Published:** 2009-09-01

**Authors:** Gabriel Iturriaga, Ramón Suárez, Barbara Nova-Franco

**Affiliations:** Centro de Investigación en Biotecnología-UAEM, Av. Universidad 1001, Col. Chamilpa, Cuernavaca 62209, Morelos, Mexico; E-Mails:rsuarez@uaem.mx (R.S.);bnovafranco04@gmail.com (B.N.-F.)

**Keywords:** abiotic stress, anhydrobiosis, arabidopsis, dehydration, drought tolerance, osmoprotectant, sugar sensing, transgenic plants, trehalose

## Abstract

Trehalose is a non-reducing disaccharide formed by two glucose molecules. It is widely distributed in Nature and has been isolated from certain species of bacteria, fungi, invertebrates and plants, which are capable of surviving in a dehydrated state for months or years and subsequently being revived after a few hours of being in contact with water. This disaccharide has many biotechnological applications, as its physicochemical properties allow it to be used to preserve foods, enzymes, vaccines, cells etc., in a dehydrated state at room temperature. One of the most striking findings a decade ago was the discovery of the genes involved in trehalose biosynthesis, present in a great number of organisms that do not accumulate trehalose to significant levels. In plants, this disaccharide has diverse functions and plays an essential role in various stages of development, for example in the formation of the embryo and in flowering. Trehalose also appears to be involved in the regulation of carbon metabolism and photosynthesis. Recently it has been discovered that this sugar plays an important role in plant-microorganism interactions.

## 1. Introduction

Water is a key element for life, however some organisms have evolved an amazing adaptation that allows them to survive under complete dehydration conditions for months or years, until water is present again, at which time they resume their metabolism and growth. Anhydrobiosis (“life without water”) is found throughout all biological domains, for example in several species of eubacteria, archea, some fungi, certain invertebrate species and “resurrection plants”. All these organisms accumulate the disaccharide trehalose [1]. Investigations into desiccation tolerance began in the XVII century when Leeuwenhoek, using a microscope, observed certain organisms in the dust from his house roof, which came alive again after being in contact with water [2]. Years later in 1832, trehalose was discovered by Wiggers among grains of rye infested with fungi. In 1858 Berthelot obtained a disaccharide from insect larvae named “trehala manna” and named it trehalose [3]. Koch and Koch in 1925 noted the presence of a glassy substance in a solution made from yeast extract, which had been stored for a long period of time. This sugar can be detected by the use of trehalase, which breaks trehalose down into two glucose residues [4].

## 2. The Multiple Roles of Trehalose

For many years trehalose was considered to be a rare sugar because it had only been isolated from resurrection plants, yeast and the larvae of certain insects. Today this sugar has been found in a wide number of organisms, although in many cases in low concentrations. Trehalose is a non-reducing disaccharide in which two glucose molecules are joined together by a glycosidic α-(1-1) bond.

The physicochemical properties of trehalose make this molecule an exceptional sugar. The glycosidic bond that joins the two hexose rings has low energy (1 kcal/mol), which makes it a very stable structure in comparison with sucrose; the latter non-reducing sugar displays a high-energy bond (27 kcal/mol). Trehalose is not easily broken into its two glucose molecule components, except in the presence of trehalase that can be found in cellular cytoplasm with a neutral pH or within vacuoles with a pH of 4.5 [5].

Because trehalose is a non-reducing sugar, it is also known for not participating in the Maillard reactions that cause food browning, which consist of a series of complex changes involving amines, amino acids, peptides or proteins and the carbonyl groups of reducing sugars. This makes trehalose an attractive molecule for industrial applications, as it also shows 45% sweetness, when compared to sucrose [6,7].

Three possible mechanisms have been put forward to explain how trehalose protects biomolecules: water replacement, glass formation and chemical stability [8]. These mechanisms are not mutually exclusive and may all contribute to the stabilizing effects of trehalose. The water replacement theory suggests that all biological macromolecules are normally stabilized by water, which forms hydrogen bonds around the molecules (hydration layer). It has been proposed that trehalose in solution protects biological structures against water removal that takes place during dehydration or freezing, replacing the water molecules in the hydration layer [9]. This mechanism would help to stabilize the biomolecules and inhibit their irreversible denaturation. The glycosidic link between the two d-glucose residues of trehalose displays great flexibility in comparison with other disaccharides. This property may allow trehalose to interact with other irregular polar groups of different macromolecules [10]. Trehalose is the only sugar that forms amorphous, non-hygroscopic crystals and is stable at high temperatures, even when the crystal is in an anhydrous state [1]. This allows trehalose to form a vitreous state and to continue intact for longer times at extreme temperatures than other sugars. The biomolecules can remain indefinitely (months or years) in this vitreous state, allowing them to return to their original structure after being rehydrated. Besides, the temperature for transition to the vitrification state is higher in trehalose than in other common sugars [11].

## 3. Biotechnological Applications of Trehalose

The properties of trehalose have made it a valuable biotechnological product with diverse applications, some of which have been developed for commercial use [12–14]. So far, the most common and promising uses for this disaccharide are:

*Protector of enzyme activity*: Trehalose can be used to store thermolabile enzymes such as DNA polymerase, restriction enzymes and DNA ligase at ambient temperature [15].
*Stabilizer and protector for complex molecules*: Unstable molecules such as antibodies can be dehydrated at room temperature or 37 °C in the presence of trehalose, maintaining their activity after various months in storage [8].
*Foods additive*: Trehalose can be used in dried or processed foods such as fruits and vegetables, in order to preserve aromas and their organoleptic properties; it should also be pointed out that this disaccharide is not toxic and it is already consumed as part of the human diet, as it is present in bread, honey, mushrooms, wine, beer, etc [16].
*Preserver of cells, tissues and organs*: Cells, tissues and even organs can be preserved for months in the presence of trehalose, either dried or frozen, improving shelf-life in comparison with other substances [17,18].
*Improvement of flower shelve-life*: Addition of 50 to 100 mM trehalose to tulips and gladioli increases their shelf-life in a vase after cutting, as it apparently avoids transpiration [19,20].
*Selection marker in the production of transgenic plants*: The *AtTPS1* gene of *A. thaliana* encodes the TPS1 enzyme, which confers glucose insensibility to seeds and tissues of plants overexpressing this gene when cultivated under tissue-culture conditions [21]. Germination and differentiation in wild type plants are inhibited by glucose, thus *AtTPS1* can be used as a selectable marker gene during the process of plant transformation, using this monosaccharide as a selective agent [22].
*Cosmetics industry*: Trehalose traps and reduces bad odors emitting from human skin by up to 70%, making it a useful additive for facial or body creams and for deodorants [7].
*Possible medical uses*: The role of trehalose in reducing the symptoms in illnesses such as Huntington’s chorea and in osteoporosis has been explored. In the former, trehalose prevented the formation of polyglutamine protein in the brain [23] and in the second study, the consumption of trehalose was found to reduce the degeneration of bones in female rats whose ovaries had been removed. However, the mechanisms involved in these uses are not fully understood [7].

## 4. Transgenic Manipulation of Trehalose Metabolism

The first attempts to obtain transgenic plants that accumulate trehalose were undertaken for tobacco employing the *E. coli otsA* gene or *ScTPS1* from yeast (Table 1). The resulting plants displayed improved drought tolerance [24,25]. Tomato and potato plants expressing the *TPS1* gene have also been obtained and showed drought tolerance [26,27]. However, in all these cases, the plants exhibited morphological and growth abnormalities, probably due to the accumulation of T6P.

With the aim of avoiding the pleiotropic effects of T6P, a gene construction comprising the translational fusion of *OtsA* and *OtsB* was expressed in rice [28]. The plants displayed a normal phenotype and were tolerant to drought, salinity and cold. In a similar case, a yeast chimaeric gene coding for the TPS and the carboxy-terminal region of the TPP, when expressed in Arabidopsis resulted in plants without morphological alterations, which were tolerant to drought, salinity, freezing and heat [29]. Recently this bifunctional TPS-TPP enzyme led to alfalfa plants that showed improved tolerance to multiple abiotic stresses, however if the constitutive 35S promoter was used the plants were moderately stunted; in contrast, expression with the RD29A stress-inducible promoter led to larger plants with a significant increase in biomass [30].

Interestingly, transgenic Arabidopsis plants overexpressing the homologous *AtTPS1* gene displayed drought tolerance [21]. Contrary to the observation in other plants transformed with *TPS* genes from *E. coli* or *S. cerevisiae*, the overexpression of *AtTPS1* did not provoked any abnormalities in plants, with the exception of flowering retardation for approximately one week. The low amount of trehalose accumulated in the transgenic plants was not sufficient to explain its role as osmoprotector, however alterations in the expression of important signaling and stress-response genes may explain this phenotype [21,31].

## 5. Evolution of Trehalose Biosynthesis

**Figure 1.**
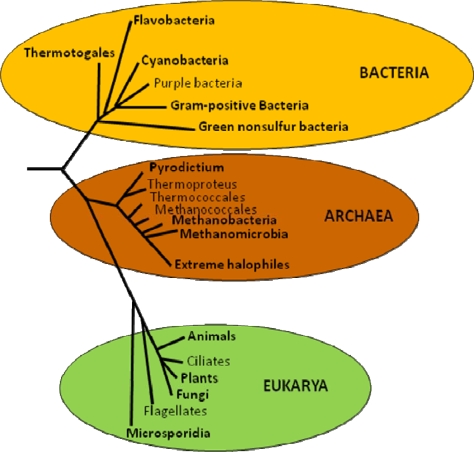
Evolutionary history of trehalose biosynthesis. The trehalose biosynthetic pathways are present in the three domains of life tree. Phyla in bold letters show organisms that have at least one of the five routes of trehalose biosynthesis.

Trehalose is widely distributed throughout the biological world (Figure 1). It is common in yeast and fungi in spores, fruit bodies and vegetative cells [32]. For example, it has been reported that trehalose represents 7% of the dry weight of *Dictyostelium mucoroides* spores and that the ascospores of *Neurospora tetrasperma* accumulate up to 10% of trehalose. It is also found in high concentrations in bread and beer yeast, where the levels of trehalose depend on the age of the cells, as well as their stage of growth and nutritional state [33]. In yeast, it can be employed as carbon storage [34] and as an adaptive response to various types of abiotic stresses [35,36]. Trehalose is also found in non-vascular plants such as the lycophyte *Selaginella lepidophylla* [37], in higher plants such as Arabidopsis [38] and in some “resurrection” grasses [39].

Trehalose is also present in mycobacteria and corynebacteria, where it plays a structural role in the cell wall. In many bacteria, trehalose also plays a role in adaptive response to osmotic stress and to extreme temperatures [40,41].

In the animal kingdom, trehalose was first reported in insects, where it is present in the hemolymph of larvae or pupae [42,43]. Among adult insects, the levels of trehalose decline rapidly during flying, indicating that this disaccharide plays a role as energy source [44]. The disaccharide was found in nematode eggs of *Ascaris lumbricoides,* where it can be as high as 8% of dry weight [43]. It is also common to find considerable levels of this sugar in certain stages of development of anhydrobiotic organisms, such as the tardigrade *Echiniscus blumi* and the crustacean *Artemia salina* [6].

One of the fundamental challenges that is faced by most organisms, from the first cells which resided in the primitive seas has been the potential to survive environmental changes, mainly, extreme temperatures, salinity and dehydration [45,46]. These organisms evolved two different strategies to counteract abiotic stress: in certain species which live in extreme environments, for example thermophiles and halophiles, their metabolic capacities underwent changes for optimum enzymatic activity and membrane stability in high temperatures or salinity, correspondingly [47].

**Figure 2.**
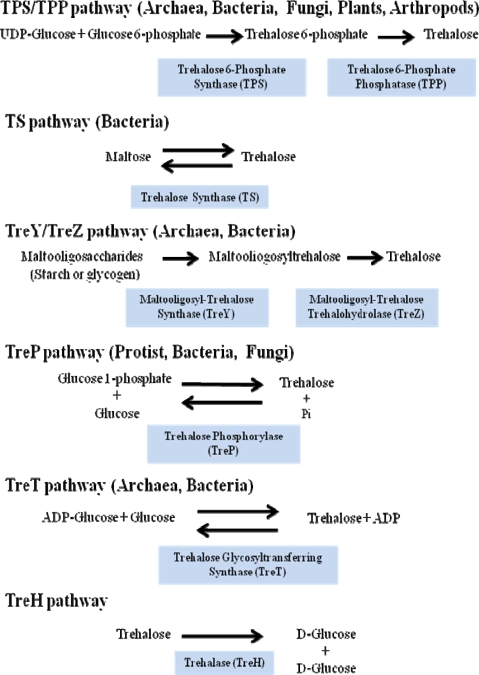
Trehalose biosynthetic and catabolic pathways and distribution in eukaryotes and prokaryotes.

Other microorganisms, when exposed to extreme conditions become adapted and struggle against stress by means of biosynthesizing osmotically active compounds, for osmoprotection and thermoprotection. Among these compounds are the polyols such as mannitol and sorbitol, certain amino acids such as proline and glutamic acid; quaternary salts such as glycine betaine and disaccharides such as sucrose or trehalose [48]. There are five pathways for trehalose biosynthesis present in the three domains of the tree of life (Figure 2):

- The most common and the best studied route among different species involves the enzyme trehalose-6-phosphate synthase (TPS), which catalyses the transfer of glucose by means of UDP-glucose to glucose-6-phosphate, leading to trehalose-6-phosphate (T6P). In a second stage trehalose-6-phosphate phosphatase (TPP) catalyzes the hydrolysis of the phosphate group from the intermediate disaccharide to generate trehalose [49]. This TPS-TPP route is found in a variety of organisms; for example insects [50], plants such as the “resurrection” plant *Selaginella lepidophylla* and *Arabidopsis thaliana* [38,51], *Escherichia coli* [40] and *Saccharomyces cerevisiae* [35].
- Trehalose synthase (TS) catalyses an intramolecular arrange of maltose, in order to convert the glycosidic bond α-(1–4) of this disaccharide to the α-(1-1) trehalose bond [52]. This enzyme is found in several organisms such as *Pimelobacter* sp, *Pseudomonas syringae* and *Thermus caldophilus.*
- The TreY-TreZ pathway is present in some bacteria where the conversion of maltooligosaccharides present in starch, are broken down to trehalose. The thermophilic archaebacteria belonging to the *Sulfolobus* genus, as well as *Arthrobacter* sp Q36 and *Rhizobium* sp M-11, display amylolytic activity which leads to trehalose. This is a two-step pathway involving maltooligosyl-trehalose synthase (TreY) catalyzing the conversion of maltodextrines to maltooligosyl-trehalose and subsequently the maltooligosyl-trehalose trehalohydrolase (TreZ) breaks this intermediate to generate trehalose [53].
- Trehalose phosphorylase (TreP) has been reported in *Agaricus bisporus*, *Catellatospora ferruginea, Euglena gracilis* and *Flammulina velutipes*. TreP catalyzes a reversible reaction *in vitro*, which hydrolyzes trehalose and transfers a glucose molecule to the inorganic phosphate, to form glucose-1-phosphate and release free glucose [54]. This reaction can go in one direction or another, depending on the species [55].
- TreT is the trehalose glycosyltransferring synthase, which results in trehalose formation from ADP-glucose and glucose. This reaction is very similar to that described for UDP-glucose and glucose-6-phosphate, but varies because the synthesis occurs in a single reaction and the products are trehalose and ADP. This reversible reaction has been detected in various organisms, such as *Thermococcus litoralis* and S*ulfolobus solfataricus* KM1 [56].

Trehalase is responsible for the catabolism of trehalose, leading to two α-d-glucose molecules. This enzyme is present in a great number of organisms and its function is to mobilize trehalose when it is no longer required or when it is used to provide energy and/or substrates to the cell. In order to obtain detectable amounts of trehalose from Arabidopsis, tobacco or potato, Validamycin A has been employed to inhibit trehalase [57].

With the aim of identifying the biosynthesis genes for trehalose in all completely sequenced genomes, a detailed analysis was undertaken using database searches for TPS and TPP sequences [58]. It was discovered that eubacteria possess five routes for the biosynthesis of trehalose and archeae has four routes, lacking the TS pathway. Fungi, plants and metazoa only possess the TPS/TPP pathway. Interestingly, among vertebrates there are no genes for the biosynthesis of trehalose, but the trehalase gene is present. On the other hand, the TPS and TPP domains in prokaryotes are generally found in operons, whereas in eukaryotes these domains have undergone parallel evolution as well as duplication [58]. In Arabidopsis genome there are 11 TSP genes, subdivided in Class I (*AtTPS1–5*) and Class II (*AtTPS5–11*) depending on whether they display most similarity to yeast *TPS1* or *TPS2* [59]. However, this plant accumulates negligible levels of trehalose. It has been shown that the only protein with enzymatic activity is AtTPS1, which raises the question concerning the function of the other 10 genes, which so far is unknown. A third gene family involved in trehalose metabolism in Arabidopis is Class III (*AtTPPA-J*), whose members display significant homology to TPP enzymes [60]. Class I and II encoded proteins exhibit a fused TPS domain and seem to be monophyletic compared to yeast TPP. Class III proteins were probably recruited in plants after the divergence from fungi since they are not present in this kingdom as single domain proteins. It is interesting that Class III proteins are closely related to *Mycobacterium* and it is tempting to speculate that they were recruited from bacteria (for instance, by endosymbiosis) by an ancestor of contemporary plants [58]. It was found by bioinformatics analysis that all *TPS* plant genes (Class I, II and III) are under selection pressure suggesting that all of them have a particular function, which could probably be related to other processes not necessarily related to osmoprotection [58].

Analysis of Arabidopsis, rice and poplar genomes showed that they contain large families of *TPS* and *TPP* genes [61]. Arabidopsis is atypical in having four Class I *TPS* genes, three of which (*AtTPS2–4*) encode unusual short isoforms of TPS that appear to be found only in members of the Brassicaceae family. Both Class I and II are represented in the genomes of chlorophyte algae and non-flowering plants. Therefore, it is suggested that plant TPS genes are very ancient, possibly pre-dating the divergence of the streptophyte and chlorophyte lineages [61].

To approach the function of Arabidopsis Class II proteins, the promoter of these seven genes was fused to reporter genes. Gene induction after incubating transgenic Arabidopsis plants demonstrates a differential tissue-specific expression and responsiveness to carbon availability and hormones. None of the Arabidopsis Class II proteins displays significant TPS or TPP activity when expressed in yeast, thus consistent with a regulatory rather than metabolic function for these proteins [62]. It was speculated that Class II TPS enzymes could be monitoring of T6P levels, thereby acting as T6P sensors of carbohydrate availability and triggering tissue-specific responses.

## 6. Trehalose-6-Phosphate as a Signaling Molecule

The first evidence that trehalose metabolism was involved in other functions besides osmoprotection, came from work on the yeast *tps1* mutant that is unable to grow in glucose but can be rescued if grown in a non-fermentable sugar as galactose, suggesting that TPS has a regulatory role in glycolysis, where some glucose-6-phosphate is converted into trehalose as a mechanism to restrict glycolytic flux [63]. The yeast double mutant *tps1hxk2Δ* lacks both TPS and Hexokinase 2 (HXK2), and can be rescued by growing in the presence of glucose. The main function of HXK2 is to add phosphate moieties to glucose as a gate to glycolysis. It has been demonstrated that T6P inhibits yeast HXK2 activity so it is likely that this metabolite regulates glycolysis by modulating the flow of phosphorylated sugars towards this pathway [63,64].

In plants, the isolation of an Arabidopsis *tps1* mutant displaying an embryo-lethal phenotype strongly suggested that trehalose plays a key role as a signal activating development [65]. It was possible to partially rescue embryonic development *in vitro*, by reducing the supply of sucrose, but not by adding T6P, possibly due to the lack of transport into the cell of this molecule. Also, the *tps1* mutant plastids accumulate large starch granules persistent during seed development, in contrast with wild type plastids where starch accumulation is transient. This is paralleled by the thickening of *tps1* cell walls, suggesting that *AtTPS1* in Arabidopsis may play a major role in coordinating cell wall biosynthesis and cell division, with cellular metabolism during embryo development [66]. Another interesting example of trehalose metabolism affecting plant development is the *RAMOSA3* maize mutant that encodes a defective TPP, indicating that T6P controls inflorescence architecture [67].

Interestingly, the *Caenorhadbitis elegans gob-1* mutant results in early larval lethality in part due to a blocked intestinal lumen and consequent starvation [68]. It turns out that *GOB-1* encodes a TPP homologue and it is suggested that T6P built up is possible causing a toxic effect.

*AtTPS1* is also essential for Arabidopsis to develop from its vegetative state to flower. The *tps1* mutant line transformed with *AtTPS1* under a dexamethasone-inducible promoter was able to continue its normal development to mature plants in the same way as the wild type plants, however if feeding with the inductor was stopped before flowering, it was unable to develop floral buds [69].

Other lines of evidence suggesting that AtTPS1 enzyme in Arabidopsis is involved in the glucose signaling pathway, stems from experiments showing that the overexpression of the *AtTPS1* gene provokes glucose and abscisic acid (ABA) insensitivity in Arabidopsis seedlings [21,31]. In these studies, *AtTPS1* expression in wild type plants is inhibited in the presence of glucose, however this inhibition is overcome in a *HXK1* knock-out genetic background, suggesting that AtTPS1 participates in the sugar signaling pathway mediated by the HXK1 sugar sensor [70]. In an independent work, it was shown that the overexpression of *E. coli OtsA* (bacterial TPS) gene in Arabidopsis was unable to counteract the inhibition caused by glucose [71]. This apparent contradiction could be explained after comparison of OtsA and AtTPS1 deduced protein sequence, where the latter one possesses amino- and carboxy-terminal ends, in addition to the catalytic domain that is homologous to OtsA. It seems possible that the amino-terminal end may have a regulatory function, because when deleted the enzymatic activity of AtTPS1 is increased by up to 40 times [72]. Besides, phosphorylated class II plant TPS are known to interact with 14-3-3 phosphoprotein, possibly to mediate responses to signals that activate SnRK1 [73].

Another set of data supporting a signaling role for trehalose metabolism, show that *AtTPS1* regulates the transcription of *ABI4*, which in turn acts as a transcriptional repressor of photosynthesis genes [21,31,74]. Arabidopsis seedlings grown on trehalose-containing medium display increased expression of the starch synthesis gene *ApL3* encoding the large subunit of ADP-Glc pyrophosphorylase (AGPase, the first enzyme in starch synthesis) and down-regulation of genes involved in starch breakdown, such as SEX1, which encodes a glucan-water-dikinase and the β-amylase gene BMY8/BAM3. Both processes are apparently mediated by *ABI4* [75].

**Figure 3.**
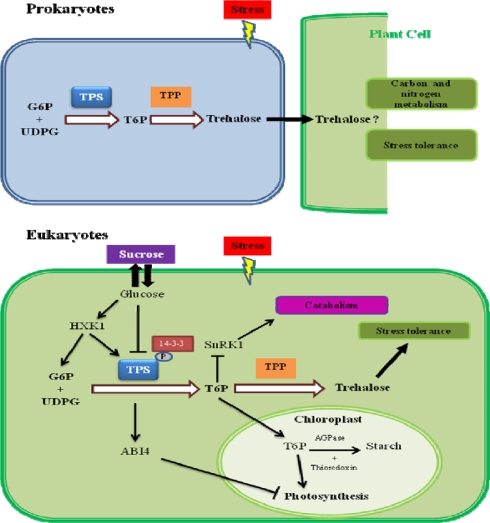
The role of trehalose pathway in prokaryotes and eukaryotes. Trehalose in prokaryotes dramatically accumulates in osmotic or thermal stress conditions; it can signal from the bacteria to the plant cell stress tolerance and nitrogen and carbon metabolism. In plants, trehalose 6-phosphate (T6P) plays a central role regulating sugar metabolism and plant development. Glucose and trehalose are also important keys to several signaling and regulatory pathways and integrate external cues to adapt cells to abiotic stress, growth and development. It seems that TPS1 and ABI4 are part of the HXK1 signaling pathway. Other molecular actors in this network are the 14-3-3 proteins, which are known to interact with phosphoserine in diverse proteins including TPS; and SnRK1, which signals catabolism in starvation conditions, and is countered by T6P to induce anabolism. Thus, an important role of the trehalose biosynthesis pathway in higher plants would be the synthesis of small amounts of T6P and/or trehalose signaling molecules rather than accumulation of this latter as an osmoprotective compound.

It has also been demonstrated that T6P positively regulates photosynthesis and starch synthesis in plastids [76–78]. This latter process is mediated by activation of ADP-glucose pyrophosphorylase (AGPase) *via* posttranslational redox modification by thioredoxin [79]. The response is dependent on expression of SNF1-related kinase (SnRK1). SnRK1 was shown to affect the transcript abundance of approximately 1,000 genes in Arabidopsis, playing a central role in the response to starvation [80]. Recent evidence demonstrates that T6P functions as an inhibitor of SnRK1 and analysis of microarray data showed up-regulation by T6P of genes involved in biosynthetic reactions, which are normally down-regulated by SnRK1 [81]. Thus, all these molecular players act in concert with T6P, trehalose, and AtTPS1 to promote seedling development, photosynthesis, carbohydrate regulation, and stress adaptive responses (Figure 3).

## 7. Involvement of Trehalose in Pathogenesis and Symbiosis

For some time it has been observed that trehalose plays an important role in both beneficial and pathogenic interactions between plants and microorganisms. Initially, the role played by trehalose during the process of infection came from studies where gene disruption of the *TPS1* gene in *Candida albicans* caused a decrease in the pathogenic infection, drastically affecting the development of hyphae [82]. On the other hand, *TPS2* gene knockout generates hyperacumulation of T6P, thermosensibility and cellular death, after a few hours of growth at 44 °C [83]. This mutant also affects the virulence at a systemic level in animals, as it significantly drops infection in mice.

In *Mycobacterium tuberculosis* the TPS-TPP, TreS and TreYZ pathways for trehalose biosynthesis are present in the genome, and the genes encoding the three routes were individually deleted and the effect of each one in terms of the viability of infection were determined. The TPS-TPP pathway turns out to be essential for pathogen attack and infection development in mice. The TreYZ pathway did not significantly affect the development of the pathogen and the virulence in mice, whereas gene deletion of the TS pathway altered the later stages of pathogenesis, significantly retarding the time of death, so that chronic illness had time to take hold [84].

Mutation of *OtsA* gene in the rice pathogen *Magnaporthe grisea*, caused a significant drop in plant infection apparently by inadequate penetration of the hyphae into the vegetative cells due to turgor loss [85]. Additionally, it is known that trehalase is induced in Arabidopsis infected with the fungi *Plasmodiophora brassicae* [86]. However, there is not yet a clear mechanism to explain the role of trehalose in pathogenic interactions.

Trehalose is also known to be present in certain symbiotic interactions such as endomycorrhizae and ectomycorrhizae and in legume nodules [87,88]. As nodules grow older, the nitrogenase decreases but the proportion of trehalose in the bacteroids increases. This could be the reason why the number of bacteria per nodule remains constant in senescence since trehalose may serve as a carbon source for the growth and survival of *Bradyrhizobium japonicum* [89]. Besides, trehalose biosynthesis in nodules seems induced by the low oxygen tension of microaerobic environment [90].

In order to study the role played by trehalose accumulated in rhizobia during symbiosis with legumes, a *Rhizobium etli* strain was generated that overexpressed the *OtsA* gene, and a mutant in the homologue gene, in order to analyze their effect on the survival of free-living bacteria and in symbiosis with *Phaseolus vulgaris* [91]. First of all, the results showed that the free-living bacteria with increased trehalose content were tolerant to osmotic stress and in contrast, the *otsA^−^* mutant was osmosensitive. Also, the bean plants inoculated with the *OtsA* overexpressing strain had a higher number of nodules and nitrogenase activity, greater biomass, increased drought tolerance and showed a significant increase in grain yield, under normal watering or stress conditions.

The stronger evidence suggesting that trehalose has a signaling role in plant-bacteria interactions comes from a transcriptomic analysis of plant nodules in symbiosis with *R. etli* overexpressing its own *TPS* gene [91]. The genomic data revealed induction of several genes involved in stress tolerance, and carbon and nitrogen metabolism, thus suggesting that trehalose plays a role as a signal molecule possibly exported from rhizobia to the nodule cells of the legume plant (Figure 3). So far there is no evidence of how bacterial trehalose could regulate these plant genes, and whether this disaccharide is the signal itself traveling to the plant or if there is another intermediate metabolite that triggers gene transcription in plant nodules (Figure 3).

Recently, a similar effect concerning drought tolerance and increase in biomass was observed in maize plants, inoculated with genetically modified *Azospirillum brasilense* that overaccumulated trehalose [92]. Overall, these plant-microbe studies strongly suggest that engineering soil bacteria with trehalose metabolism genes is a potential tool for improving drought tolerance, biomass and yield in economically important crops.

## 8. Conclusions

Trehalose is one of the major osmoprotectants found in Nature and its biosynthesis capacity is present in most organisms, except vertebrates. However, this disaccharide accumulates only in a few species, known as anhydrobionts, among the three life domains. In rice and Arabidopsis the *TPS* and *TPP* gene families are represented by several gene-copies although trehalose is hardly detected higher plants, where sucrose is the most abundant sugar. During evolution desiccation tolerance in vascular plants was mainly restricted to the seed stage and trehalose biosynthesis acquired other important roles such as signaling carbohydrate usage during development and plant growth. Most of the studies have focused on T6P as the signaling molecule but the role of trehalose itself cannot be excluded completely as yet. Transgenic plants expressing trehalose biosynthesis genes have led to a stress tolerance phenotype, however there is little trehalose accumulation and several lines of evidence strongly suggest that T6P is activating many genes downstream probably by inhibiting SnRK1. This might be the case as well in bacteria engineered for trehalose accumulation to interact with crops, thus becoming stress tolerant and with an increased yield. Understanding how exactly trehalose and T6P interact with putative targets and activate metabolic and stress pathways is far from complete and more research must be conducted which in turn could impact agriculture.

## Figures and Tables

**Table 1.** Transgenic plants expressing trehalose biosynthetic genes.

**Used gene**	**Origin**	**Promoter**	**Transformed plant**	**Morphological alterations**	**Tolerance**	**Reference**
***TPS1***	Yeast	35S	Tobacco	Yes	Drought	24
***OtsA*^a^**	*E. coli*	35S	Tobacco	Yes	Drought	25, 28
***TPS1*^b^**	Yeast	35S	Potato	Yes	Drought	26
***TPS1***	Yeast	35S	Tomato	Yes	Drought, salinity	27
***TPS1***	Arabido psis	35S	Arabidopsis	Flowering delay	Drought	21
***OtsA-OtsB*^c^**	*E. coli*	ABRC1-actin1^d^	Rice	No	Drought, salinity, cold	28
***TPS1-TPS2*^e^**	Yeast	35S, RD29A^f^	Arabidopsis	No	Drought, salinity, heat, freezing	29
				No		
***TPS1-TPS2***	Yeast	35S, RD29A	Alfalfa	Stunted	Drought, salinity, heat, freezing	30
				Larger		

^a^ *OtsA*, *Escherichia coli* trehalose-6-phosphate synthase (*TPS*);

^b^ *TPS1*, *Saccharomyces cerevisiae TPS*;

^c^ *OtsB*, *Escherichia coli* trehalose-6-phosphate phosphatase (*TPP*);

^d^ ABRC1-actin1, abscisic acid-inducible promoter coupled with a minimal actin1 promoter;

^e^ *TPS2*, *Saccharomyces cerevisiae TPP*;

^f^ RD29A, stress inducible promotor.
